# Supplementary material for: Identifying Risk Genes and Interpreting Pathogenesis for Parkinson’s Disease by a Multiomics Analysis
Source: Genes (Basel). 2020 Sep 21;11(9):1100. doi: 10.3390/genes11091100 (PMC7563773; doi:10.3390/genes11091100)
Supplement: Supplementary file 1 [file genes-11-01100-s001.pdf]

genes

MDPI

Supplementary Material

Supplementary Table 1. Summary of biological function items by GO enrichment.

Name	GeneRatio	BgRatio	p-value
Axon part	14/236	373/19659	2e-04
Juxtaparanode region of axon	3/236	10/19659	2e-04
Neuron to neuron synapse	13/236	342/19659	3e-04
GABA-ergic synapse	6/236	75/19659	3e-04
Asymmetric synapse	12/236	320/19659	5e-04
Axolemma	3/236	15/19659	7e-04

Supplementary Table 2 Summary of risk genes for independent GWAS datasets screened by SMR.

ProbeID	Chr	Gene	topSNP	P_{GWAS}	FDR P_{SMR}	P_{HEIDI}
ILMN_1784428	17	*MGC57346*	rs1526126	2.04e-55	1.20e-09	9.79e-01
ILMN_1743621	17	*C17ORF69*	rs3418	4.85e-57	1.10e-08	6.57e-01
ILMN_1678235	17	*KIAA1267*	rs2532276	1.55e-52	5.55e-07	9.89e-01
ILMN_1701933	4	*SNCA*	rs1372519	4.50e-36	3.16e-06	3.68e-10
ILMN_1766165	4	*SNCA*	rs1372519	4.50e-36	3.16e-06	9.28e-10
ILMN_1680353	17	*NSF*	rs199530	8.96e-41	2.31e-05	7.17e-02
ILMN_2286783	17	*LRRC37A4*	rs241035	4.45e-57	2.00e-04	NA[1]
ILMN_1793017	4	*DGKQ*	rs11724804	7.76e-20	8.22e-04	4.64e-02
ILMN_1813685	1	*RAB7L1*	rs823114	1.06e-20	1.46e-03	6.72e-01
ILMN_1770161	4	*BST1*	rs4698412	2.01e-15	6.72e-03	9.69e-01
ILMN_2115154	7	*NUPL2*	rs858300	2.70e-15	8.37e-03	6.96e-01
ILMN_1789616	7	*NUPL2*	rs858305	3.83e-15	8.94e-03	9.89e-01
ILMN_1801205	7	*GPNMB*	rs199357	8.23e-16	1.47e-02	4.94e-01
ILMN_1739798	7	*C7ORF30*	rs156429	8.46e-16	2.48e-02	9.98e-01
ILMN_1656361	17	*LOC201175*	rs9303471	5.79e-12	3.00e-02	7.73e-02
ILMN_1680313	16	*STX4*	rs8056842	1.33e-10	3.71e-02	4.20e-01
ILMN_1814726	4	*SCARB2*	rs13122345	4.83e-12	3.71e-02	6.12e-01
ILMN_1859584	12	*HS.306876*	rs2263420	1.60e-10	4.27e-02	9.57e-01
ILMN_1696360	8	*CTSB*	rs1736081	2.96e-10	4.38e-02	1.07e-01
ILMN_2144088	8	*FDFT1*	rs1293328	7.77e-10	4.48e-02	7.89e-02
ILMN_2359742	8	*CTSB*	rs1296028	2.56e-10	4.48e-02	1.75e-01

1.NA = Not available.

Supplementary Table 3. Summary of effect value information of PD risk genes.

ProbeID	Chr	Gene	b_{GWAS}	SE_{GWAS}	b_{eQTL}	SE_{eQTL}	b_{SMR}	SE_{SMR}
ILMN_1784428	17	*MGC57346*	-0.26	0.04	0.86	0.02	-0.30	0.05
ILMN_1743621	17	*C17ORF69*	-0.26	0.04	0.50	0.02	-0.51	0.09
ILMN_1678235	17	*KIAA1267*	-0.25	0.04	-0.29	0.02	0.88	0.16
ILMN_1680353	17	*NSF*	-0.24	0.04	0.22	0.02	-1.09	0.21
ILMN_1770161	4	*BST1*	-0.14	0.03	0.58	0.02	-0.24	0.06
ILMN_1680313	16	*STX4*	-0.15	0.03	0.33	0.02	-0.45	0.11
ILMN_2286783	17	*LRRC37A4*	-0.23	0.04	-0.14	0.02	1.58	0.38
ILMN_1793017	4	*DGKQ*	-0.13	0.03	0.95	0.01	-0.14	0.03
ILMN_1790114	17	*LOC474170*	-0.24	0.04	0.13	0.02	-1.82	0.45

Supplementary Table 4. Summary of transcription-related DNAm probe enrichment in 14 functional regions.

Marks	Fold-change	P-value
TssA	1.19	9.15e-06
Promoter	1.27	1.01e-54
Tx	1.32	2.44e-27
TxWk	1.22	2.19e-09
TxEn	1.31	3.88e-28
EnhA	1.04	1.4e-1
EnhW	1.01	8.7-01
DNase	0.83	4e-04
ZNF/Rpts	0.99	1e
Het	0.66	1e-03
PromP	1.00	1e
PromBiv	0.86	2.42e-09
ReprPC	0.79	1.32e-27
Quies	0.70	1.03e-98

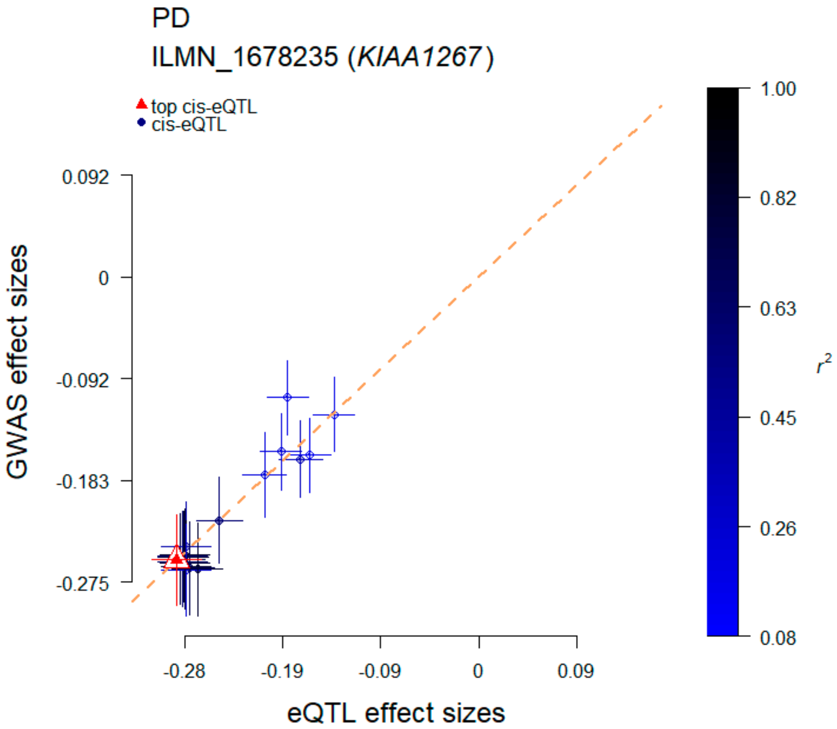

Supplementary Fig. 1 Causal effect map of *KIAA1267*. The y-axis represents the effect value of the SNP on the PD phenotype, and the x-axis shows the eQTL effect value corresponding to the SNP. The orange dotted line indicates the effect value b_{xy} corresponding to the most significant cis-eQTL, and $b_{xy} > 0$ indicates that gene expression is positive correlated with PD risk.

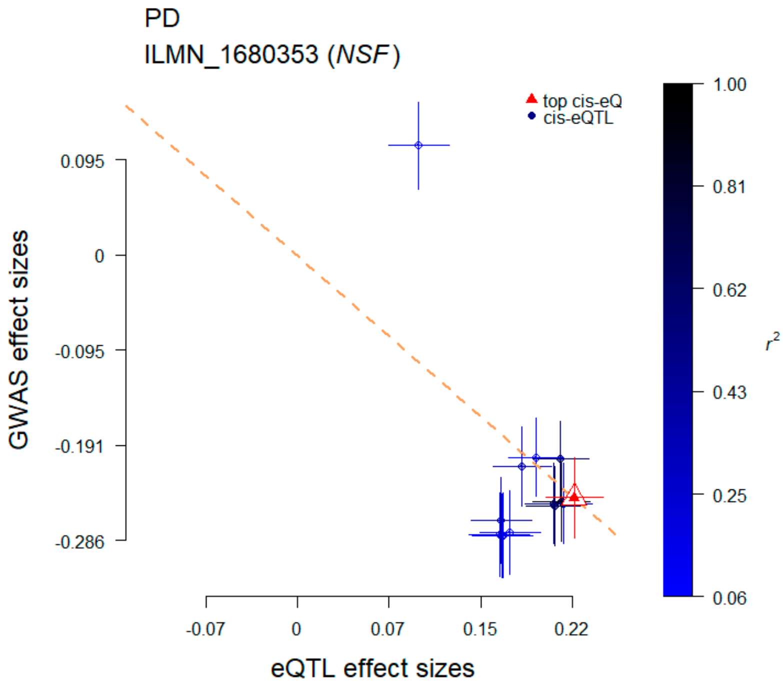

Supplementary Fig. 2 Causal effect map of *NSF* gene.

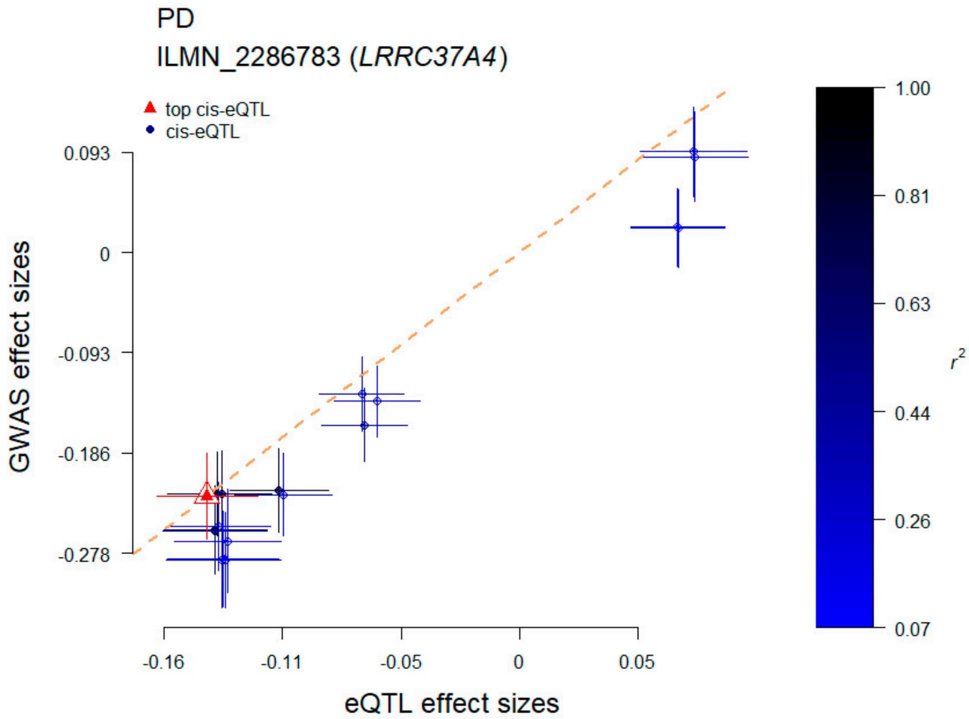

Supplementary Fig. 3 Causal effect map of *LRRC37A4*.

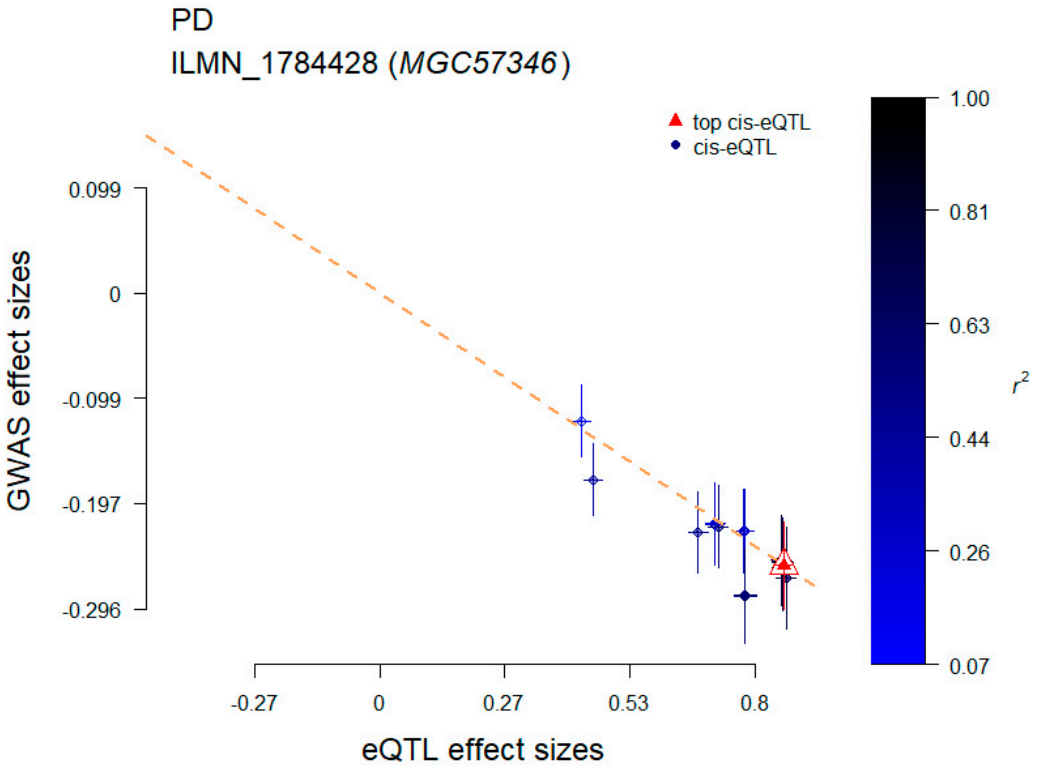

Supplementary Fig. 4 Causal effect map of *MGC57346*.

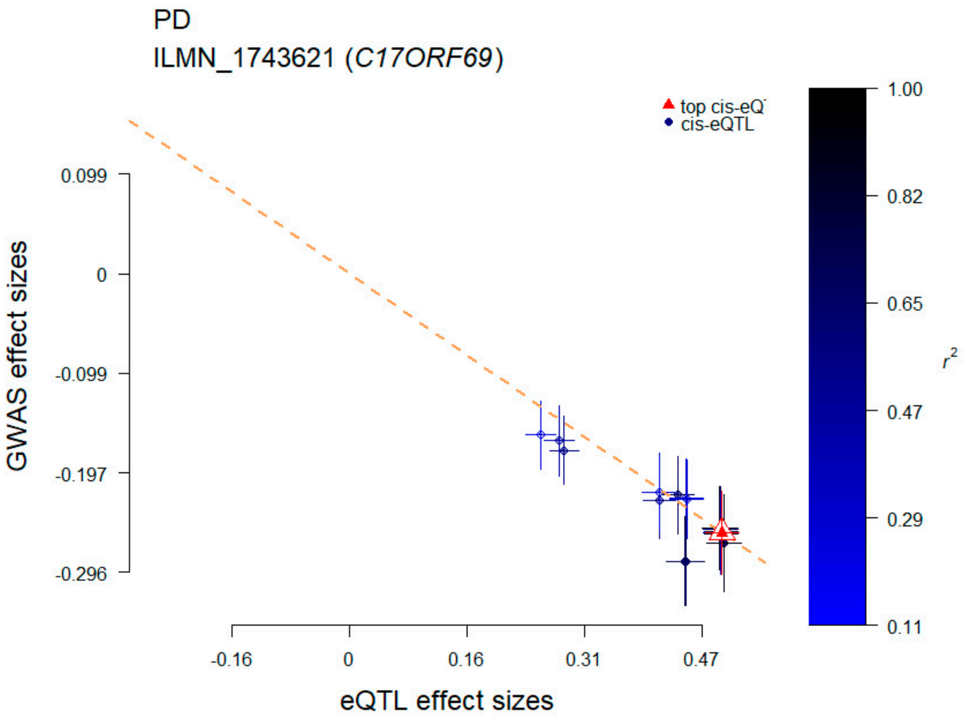

Supplementary Fig. 5 Causal effect map of *C17ORF69*.

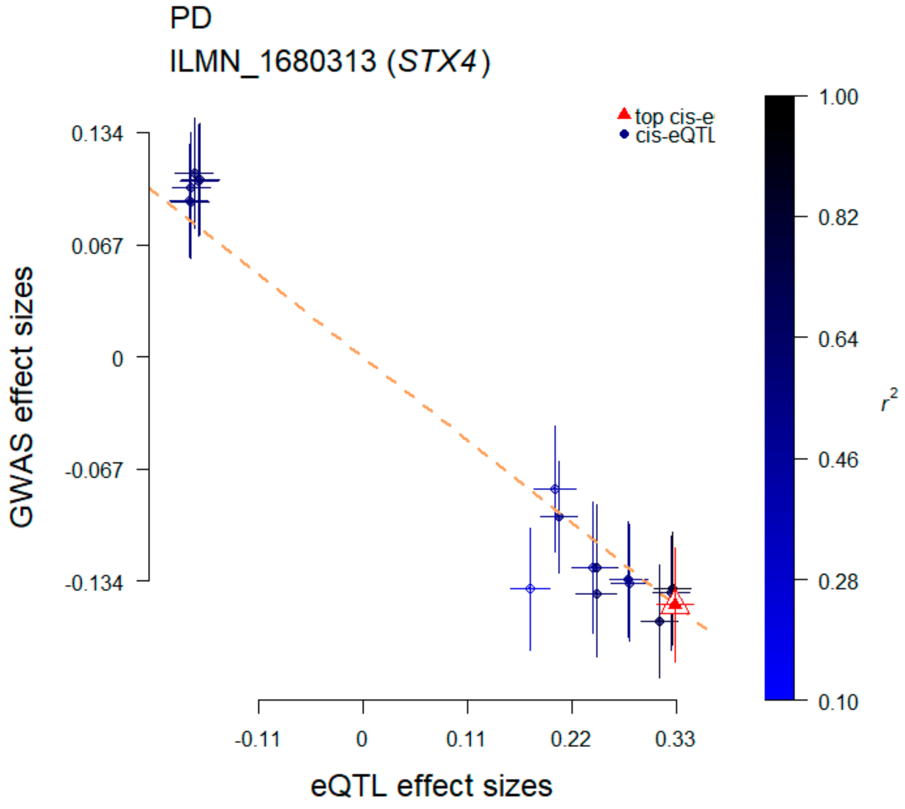

Supplementary Fig. 6 Causal effect map of *STX4*.

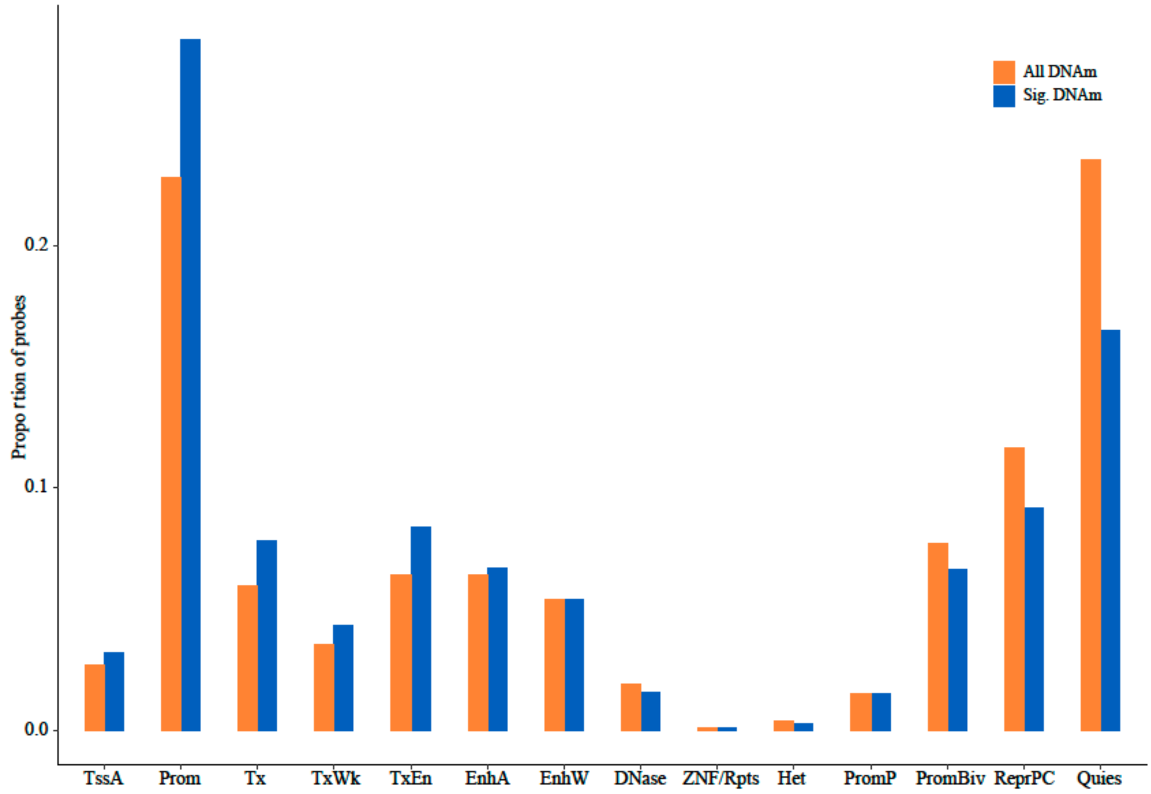

Supplementary Fig. 7 Enrichment analysis of DNAm probes in 14 main functional annotation categories. Distribution of the transcript-associated DNAm probes across the 14 functional categories in comparison to that of all DNAm probes in the data.

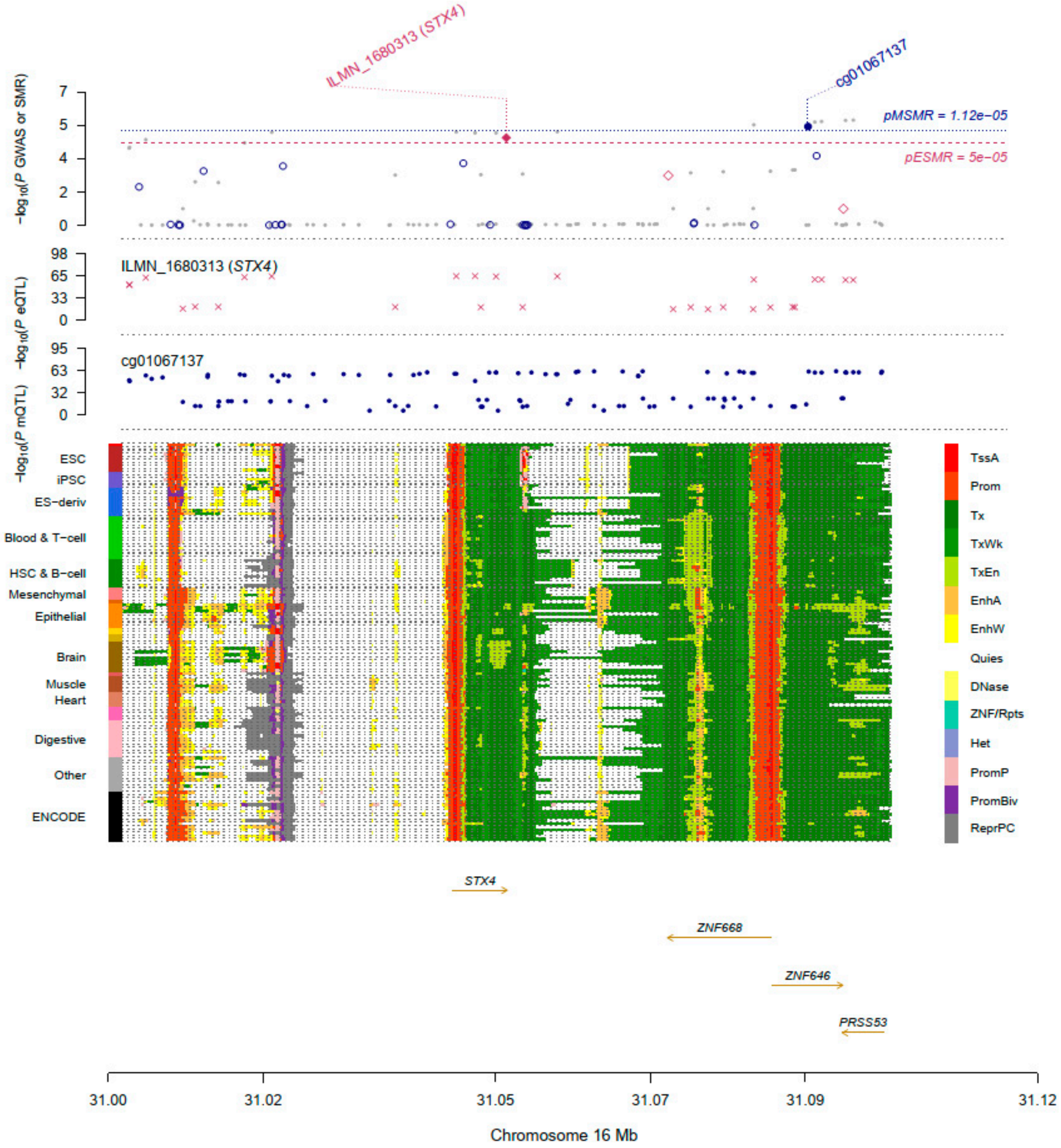

Supplementary Fig. 8 Summary of SMR correlation analysis across mQTL, eQTL and GWAS on Chr16. The figure above shows the -log10(p-values) of the SNPs from meta-analytic GWAS data. The middle two graphs are -log10(p-values) of SNPs in eQTL and mQTL, respectively. The lower diagram shows chromatin status annotation information.
